# Comparison between the effects of potassium phosphite and chitosan on changes in the concentration of Cucurbitacin E and on antibacterial property of *Cucumis sativus*

**DOI:** 10.1186/s12906-017-1808-y

**Published:** 2017-06-05

**Authors:** Moazzameh Ramezani, Fatemeh Rahmani, Ali Dehestani

**Affiliations:** 1 Department of Biology, Faculty of Sciences, Urmia University, Urmia, Iran; 2 Department of Biology, Faculty of Sciences, Urmia University, Urmia, Iran; 3 Faculty of Sciences, Genetics and Agricultural Biotechnology, Institute of Tabarestan & Sari Agricultural Sciences and Natural Resources University, Sari, Iran

**Keywords:** Antibacterial effect, Cucumber, Cucurbitacin E, HPLC

## Abstract

**Background:**

Cucurbitacins are mostly found in the members of the family Cucurbitaceae and are responsible for the bitter taste of cucumber. Pharmacological activities such as anti-bacterial and anti-tumor effects have been attributed to these structurally divers triterpens. The aim of this study was to investigate the effect of potassium phosphite (KPhi) and chitosan on Cucurbitacin E (CuE) concentration in different tissues of *Cucumis sativus*. The antibacterial effect of plant ethanolic extracts was also examined against *E.coli* PTCC 1399 and *Pseudomonas aeruginosa* PTCC 1430 bacterial strains*.*

**Methods:**

After emergence of secondary leaves, cucumber plants were divided into 4 groups (each group consisted of 6 pots and each pot contained one plant) and different treatments performed as follows: group1. Leaves were sprayed with distilled water (Control), group 2. The leaves were solely treated with potassium phosphite (KPhi), group 3. Leaves were solely sprayed with chitosan (Chitosan), group 4. Leaves were treated with KPhi and chitosan (KPhi + chitosan). The KPhi (2 g L^−1^) and chitosan (0.2 g L^−1^) were applied twice every 12 h for one day. Fruits, roots and leaves were harvested 24 h later. The ethanolic extract of plant organs was used for determination of CuE concentration using HPLC approach. The antimicrobial activity was evaluated by the agar well diffusion method. The experiments were arranged in a completely randomized design (CRD) and performed in six biological replications for each treatment. Analysis of variance was performed by one-way ANOVA and Dunnette multiple comparison using SPSS.

**Results:**

The highest level of CuE was recorded in fruit (2.2 g L^−1^) of plants under concomitant applications of KPhi and chitosan. Result of antibacterial activity evaluation showed that under concomitant treatments of KPhi and chitosan, fruit extract exhibited the highest potential for activity against *E. coli* PTCC 1399 (with mean zone of inhibition equal to 36 mm) and *Pseudomonas aeruginosa* PTCC 1430 (with mean zone of inhibition equal to 33 mm).

**Conclusions:**

KPhi and chitosan can induce production of CuE compound and increase antibacterial potential of cucumber plant extract. The application of KPhi and chitosan may be considered as promising prospect in the biotechnological production of CuE.

## Background

Plants possess powerful resources of secondary metabolites and can be used for pharmaceutical applications and development of new drugs. *Cucumis sativus* belongs to Cucurbitaceae family and is known to be rich in cucurbitacins. Cucurbitacins have become interesting subjects in science due to their medicinal and toxic properties [1]. They are usually concentrated in fruits and roots at maturity and are responsible for bitter taste of cucumber. Seeds exhibit very low concentration of cucurbitacins [2]. The diversity of cucurbitacins lies in side chain derivatives that contribute to pharmacological actions [3]. They are known according to their structural composition and designated by the letters: A, B, C, D, E, F, G, H, I, J, K, L, O, P, Q, R and S. Cucurbitacins have also been identified outside the cucurbitaceae family including members of Scrophulariaceae, Begoniaceae, Primulaceae, Liliaceae, Tropaeolaceae and Rosaceae families [2]. Various cucurbitacins are made from chemical modification of cucurbitane (19(10–9ß)-abeo-5α-lanostane) with numerous activities such as anti-inflammatory, antitumor promotion, chemopreventive, hepatoprotective, anti-microbial, anthelmintic, antifeedant and antioxidant [4]. CuE is one of the cucurbitacins and is an active secondary methabolite with inhibition of cell adhesion actions [5] and modulatory activity effect on the peripheral human lymphocytes [6]. The compound has also been found to be a strong antifeedant for the flea beetle, bilirubin–albumin binding in human plasma and with inhibitory activity on cancer cell proliferation, actin polymerization and permeability [5]. The compound also acts as agent to protect against certain diseases in plants due to its toxicity property [7, 8]. Cu E displays superior cytotoxicity due to more hydrophobicity than the other cucurbitacins [9].

Elicitors refer to chemicals that can trigger physiological and morphological responses in plants. They include abiotic such as metal ions and inorganic compounds, and biotic such as fungi, bacteria, viruses or herbivores. Elicitors application on plants causes accumulation of a range of plant defensive secondary metabolites. The elicitor signal perception leads to initiation of a signal transduction network which activates biosynthesis of transcription factors regulating plant secondary metabolism [10]. There are various elicitors as inducers of secondary metabolites such as methionine, tryptophan, methyl jasmonate (MeJA), salicylic acid, benzothiadiazole ß-glucan, chitosan and chitin oligomers [11].

The present study was conducted to determine the effect of two plant inducers including potassium phosphite (KPhi) and chitosan on CuE production in different organs of cucumber plant using HPLC approach. Administration of plant ethanolic extracts was also investigated on prevention of *E. coli* PTCC 1399 and *Pseudomonas aeruginosa* PTCC 1430 bacterial growth under inducers application. To the best of our knowledge, this is the first time that the elicitor effect of KPhi and chitosan is reported on the accumulation of CuE bioactive compound and on enhancement of antimicrobial properties of plant extract.

## Methods

### Plant growth conditions

This study was carried out in the laboratories of Genetics and Agricultural Biotechnology Institute of Tabarestan, Sari, Iran. The native *Cucumis sativus* L. seeds were obtained from Pakan Bazr Co., Isfahan, Iran and disinfected by 70% ethanol for 5 min. Seeds were washed with distilled water, dried and sown in pots filled with sterile soil mix (equal volumes of peat, perlite and coco peat). The pots were kept at greenhouse under controlled conditions with a photoperiod of 16 h of light, 70% humidity and temperature of 24–27 °C [12] and regularly fertilized with Hoagland solution [13].

### Elicitor application

When secondary leaves emerged, the plants were divided into 4 groups (1–4) and different treatments performed as follows: group1. Leaves were sprayed with distilled water (Control), group 2. Leaves were solely sprayed with 2 g L^−1^ potassium phosphite (KPhi), group 3. The leaves were solely sprayed with 0.2 g L^−1^ chitosan (Chitosan), group 4. Leaves were sprayed with combination of KPhi (2 g L^−1^) and chitosan (0.2 g L^−1^) (KPhi + chitosan). KPhi was applied to the plants as foliar spray at concentration of 2 g L^−1^ and the control plants (0 g L^−1^). For potassium phosphite (KPhi) stocks preparation, phosphorous acid (AppliChem, Darmstadt, Germany) was partially neutralized with potassium hydroxide (by gradual mixing of phosphorus acid and potassium hydroxide solution) and the PH was adjusted to 6.3 [12]. For chitosan stock preparation, chitosan was purchased from Sigma Chemical Co. Briefly, it was dissolved in 90 ml of 0.1 M acetic acid and the solution was centrifuged for 30 min. The insoluble fractions were then discarded. This procedure was performed four times. After centrifugation, the supernatant was precipitated by adjusting of its pH to 8.0 with 5 M NaOH. The precipitates were washed extensively with distilled water and then freeze-dried. The purified chitosan was dissolved in 0.1 M acetic acid (1 g chitosan/90 ml acetic acid) and the pH of the solution was adjusted to 5.0 [14]. After appearance of secondary leaves, KPhi and chitosan were applied on the plants as foliar spray every 12 h for one day. The leaves, fruits and roots were collected 24 h later and frozen in liquid nitrogen right after harvesting.

### Determination of CuE

Fresh tissue materials were kept in an oven at 40 °C for 46 h. For determination of CuE content, dried materials of *Cucumis sativus* were ground (200 mg) and separately extracted with 5 mL ethanol (95%, *v*/v) and 5 mL CHCl_3_ at room temperature. The ethanolic extract was concentrated under reduced pressure at 40 °C. The residue was suspended in water and extracted in succession with n-hexane, benzene, chloroform, ethyl acetate and water, respectively. The residual layers were evaporated under reduced pressure at 40 °C. Every layer was checked for cucurbitacins, using phosphomolybdic acid reagent (2% of phosphomolybdic acid in absolute ethanol). Then compounds expected to be cucurbitacins were mixed with an equal volume of petrol. The sample volume was reduced to 2 ml in water bath and final volume filtered through a 0.22 μm filter (Schleicher & Shuell, Germany). Extracts were streaked on preparative chromatography and repurified again by ethanol to insure purity. The CuE Standard was used to produce the standard curve. Dilutions of standard CuE were prepared in the range of 50 to 1000 ppm. All solvents were HPLC grade and obtained from Sigma Co. Ltd. (U.S.A.). The water used for HPLC was passed through a 0.22 μm filter (Schleicher & Shuell, Germany) and degassed [15].

### Chromatographic conditions

HPLC was done using a Kontron Instruments HPLC system (Herts, U.K.) consisting of two HPLC pumps (Kontron 422), an Auto sampler 465 and a Diode Array detector 440. The curves were recorded on a computer. The column was a Bio-Sil C18 HL 90-5S column (Bio-Rad, CA, U.S.A., 250 × 4.6 mm i.d., 5 μm particle diameters, 90 Å pore size). The gradient elution, with a mobile phase of acetonitrile was used: water starting at a ratio of 2:8 and ending with a ratio of 40:60 at 20 min. The flow rate was regulated on 2.0 ml/min and CuE detected by UV absorption at 229 nm. Each sample was run in triplicates [15].

### Bacterial strains

The *Escherichia coli* PTCC 1399 and *Pseudomonas aeruginosa* PTCC 1430 were obtained from Microbiology Department, Faculty of Medical Science, Babol, Iran.

### Antimicrobial activity evaluation

The antimicrobial activity of the ethanol extract was evaluated by the agar well diffusion method using Mueller Hinton Agar No. 2 (MHA) (Thermo Scientific). Briefly, bacteria were grown on MHA at 37 °C overnight; a loop full of growth was then inoculated into Mueller Hinton broth (Thermo Scientific) and incubated at 37 °C on a rotary shaker until the turbidity of the growth was equivalent to the density of 0.5 McFarland standard. The microorganism was then spread (0.1 mL) on the surface of MHA (spreading technique). Wells of uniform diameter (6 mm) were made on the solidified agar. About 25 mg/mL of plant extracts and the negative control (solvent without plant extract) were placed separately in each well. Tobramycin (15 μg/ml) was used as positive control (Thermo Scientific). Plates were then left at room temperature for 1 h to allow the solutions diffusion into the MHA and then incubated at 37 °C overnight. Finally, the inhibition zone we measured from the base of the plate resting 5–7 cm above black flat. Each test was performed in triplets and data was recorded as mean ± SD [16, 17].

### Statistical analysis

The experiments were arranged in a completely randomized design (CRD) and performed in six biological replications for each treatment. Analysis of variance was performed by one-way ANOVA and Dunnette multiple comparison using SPSS (version 23, SPSS, Chicago, IL).

## Results

**Fig. 1:**
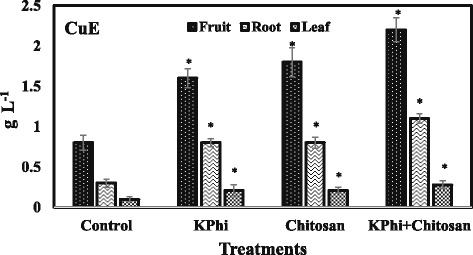
Effect of KPhi and chitosan applications on CuE (g L^−1^) level in different tissues of cucumber plants. The mean values were obtained from three independent experiments. *significantly (*p* ≤ 0.05) different according to Dunnett’s multiple comparison test compared to control. Data represent mean ± SD

The differences in contents of the CuE compound among different parts of the cucumber plant was investigated. In control plants, the CuE content in fruits reached to 2.66 and 8 fold higher than roots and leaves, respectively (Fig. 1). In fruits, the highest concentration of CuE was observed under KPhi and chitosan concomitant treatments (2.2 g L^−1^) followed by individual application of chitosan (1.8 g L^−1^) and KPhi (1.6 g L^−1^). The control fruits exhibited the lowest concentration of CuE (0.8 g L^−1^). The KPhi and chitosan introduced the same impact on induction of CuE in roots and leaves (Fig. 1). Both roots and leaves accumulated higher amount of CuE under KPhi and chitosan combination application compared to solely imposition of either KPhi or chitosan (Fig. 1).

The results obtained from antimicrobial activity evaluation showed different degrees of activity by the plant extracts (Table 1). The greatest inhibition zone was observed in KPhi and chitosan concomitant treatments against *E. coli* PTCC 1399 (with mean diameter equal to 36 mm) and *Pseudomonas aeruginosa* PTCC 1430 (with mean diameter equal to 33 mm). However, chitosan and KPhi treatments in turn showed maximum inhibition zone of 28 and 32 mm against *E. coli* PTCC 1399 and 24 and 26 mm against *Pseudomonas aeruginosa* PTCC 1430. The lowest zone of inhibition was observed in control plants against *E. coli* PTCC 1399 (13 mm) and *Pseudomonas aeruginosa* PTCC 1430 (10 mm). Results were compared with reference antibiotic (tobramycin 15 μg/ml) with zone of inhibition ranging from 15 mm (*Pseudomonas aeruginosa* PTCC 1430) to 17 mm (*E. coli* PTCC 1399).

**Table 1:** Antimicrobial activity of different ethanolic extracts of cucumber fruit (25 mg/ml) against *E. coli* PTCC 1399 and *Pseudomonas aeruginosa* PTCC 1430 bacterial growth

Treatments	Bacterial strains	
	*E. coli* PTCC 1399 (mm)	*Pseudomonas aeruginosa* PTCC 1430 (mm)
Control	13 ± 0.57	10 ± 0.06
KPhi	28 ± 0.49*	24 ± 0.89*
Chitosan	32 ± 1.59*	26 ± 1.06*
KPhi + chitosan	36 ± 1.87*	33 ± 1.49*
Tobramycin (15 μg/ml)	17 ± 0.61	15 ± 0.58

Inhibition zones presented are the average of three independent experiments. Each experiment was made in duplicate. *significantly (*p* ≤ 0.05) different according to Dunnett’s multiple comparison test compared to control. Data represent mean ± SD

## Discussion

The results of this study indicate that fruit is the most accumulator tissue among all extracts tested. Moreover, the CuE accumulation was induced by imposition of elicitors. This enhancement was inducer dependent (Fig. 1) and stronger stimulation of CuE formulation was supported by chitosan. However, exogenous addition of KPhi with chitosan resulted in a greater enhancement in biosynthesis of CuE compound. To the best of the author’s knowledge, this is the first study to present evidence concerning induction of CuE synthesis using KPhi and chitosan.

Several studies have shown that plants supplied with phosphite (Phi) elicitor display induction in synthesis of PR-proteins and secondary metabolites [14, 18, 19]. Phosphite could potentiate the synthesis of the alkaloids such as theobromine and 7-methylxanthine and the phenolics such as catechin, epicatechin, epigallocatechin, gallic acid, myricetin, p-coumaric acid, p-hydroxybenzoic acid, phloridzin, sinapinic acid, and salicylhydroxamic acid in the mango stem tissues [18].

Application of chitosan, as potential elicitor, has also been shown in accumulation of secondary metabolites in *R. graveolens* shoots [20]. In support of our findings, exposing hairy roots of *Artemisia annua* L. to chitosan (150 mg L^−1^) for six days could enhance artemisinin accumulation to 1.8 μg mg^−1^ dry wt [21]. In another study, chitosan could enhance menthol production after 12 days’ elicitation in *Mentha piperita* cell culture at concentration of 200 mg L^−1^ [22]. The enhancement in biosynthesis of isoflavonoid in *Pueraria candollei* var., oleanolic acid in *Calendula officinalis*, rosmarinic acid in *Ocimum basilicum*, indirubin in *Polygonum tinctorium*, phytoalexins in *Nicotiana tobaccum*, genistein in *Lupinus albus*, anthracene in *Rheum palmatum*, anthocyanin and phenolic acid in *Vitis vinifera*, AQ in *Rubia akane* Nakai and in *Morinda citrifolia* suspension cells has also been attributed to chitosan supply [23].

The synthesis of secondary metabolites in plants leads to antimicrobial activity against various pathogens and plays a pivotal role against pathogenic infection [14, 18, 24]. In traditional medicine, cucurbitacin-containing plants have been known for their antipyretic, analgesic, anti-inflammatory, antimicrobial and antitumor activities [25]. The pulp and the peel as well as essential oils of cucumber plant have already been shown for their antimicrobial activity against six Gram negative and Gram positive bacterial strains [26]. In this study, the zone of inhibition of bacterial growth caused by cucumber fruit extract varied from one species to another. Our result is in accordance with a study reporting antimicrobial activity of *Lagenaria siceraria* (Cucurbitaceous family) in ethanolic extract with the zone inhibition of 11 and 14 mm against *E. coli* and *Pseudomonas aeruginosa*, respectively [4]. However, the cucumber ethanolic extract showed more efficacy on *Ecoli* growth inhibition (13 mm) and less inhibitory effect on *Pseudomonas aeruginosa* (10 mm) than *Lagenaria siceraria*.

The antibacterial activity of fruit extract showed that concomitant treatments of KPhi and chitosan is more effective than individual application of either KPhi or chitosan. The higher accumulation of CuE in fruits would suggest more antibacterial activity for this part compared to roots and leaves.

## Conclusion

This research provided an insight into the medicinal potential of cucumber by focusing on the effect of individual and combination applications of two elicitors (KPhi and chitosan) on CuE production. Results reveal that concomitant application of these elicitors is an effective strategy for uplifting CuE productivity in cucumber plants. The enhancement of antibacterial activity of plant extract has also been evident against *E. coli* PTCC 1399 and *Pseudomonas aeruginosa* PTCC 1430 under imposition of both elicitors. This study, for the first time, established action of KPhi and chitosan as stimulants of CuE accumulation. From a biotechnological point of view, KPhi and chitosan combination application is of practical value for productivity increment. Further investigations must be performed to understand metabolism, absorption, distribution, and excretion of this compound. The result of this work provides information which may be useful in future researches.

## Abbreviations

CuE: Cucurbitacin E
KPhi: Potassium phosphite

## References

[1] Kupchan SM, Meshulam H, Sneden AT (1978). New cucurbitacins from *Phormium tenax* and *Marah oreganos*. Phytochemistry.

[2] Kaushik U, Aeri V, Mir SR. Cucurbitacins – an insight into medicinal leads from nature. Pharmacogn Rev. 2015;9(17):12–8. 10.4103/0973-7847.156314 PMC4441156 26009687

[3] Dinan L, Whiting P, Girault JP, Lafont R, Dhadialla TS, Cress DE, Mugat B, Antoniewski C, Lepesant JA (1997). Biochem J.

[4] Chen JC, Chiu MH, Nie R, Cordell GA, Qiu SX. Cucurbitacins and cucurbitane glycosides: structures and biological activities. Nat Prod Rep. 2005;22:386–99. 10.1039/b418841c 16010347

[5] Dong Y, Lu B, Zhang X, Zhang J, Lai L, Li D, Wu Y, Song Y, Luo J, Pang X (2010). Cucurbitacin E, a tetracyclic triterpenes compound from Chinese medicine, inhibits tumor angiogenesis through VEGFR2-mediated Jak2–STAT3 signaling pathway. Carcinogenesis.

[6] Attard E, Brincat MP, Cuschieri A (2005). Immuno modulatory activity of cucurbitacin E isolated from *Ecballium elaterium*. Fitoterapia.

[7] Bar-Nun N, Mayer AM (1990). Cucurbitacins protect cucumber tissue against infection by *Botrytis cinerea*. Phytochemistry.

[8] Gry J, Søborg I, Andersson HC (2006). Cucurbitacins in plant food. Tema Nord.

[9] Bartalis J, Halaweish FT (2005). Relationship between cucurbitacins reversed-phase high performance liquid chromatography hydrophobicity index and basal cytotoxicity on HepG2 cells. J Chromatogr B Analyt Technol Biomed Life Sci.

[10] Zhaoa J, Davis LC, Verpoorte R (2005). Elicitor signal transduction leading to production of plant secondary metabolites. Biotechnol Adv.

[11] Thakur M, Sohal BS (2013). Role of elicitors in inducing resistance in plants against pathogen infection: a review. ISRN Biochem.

[12] Mofidnakhaei M, Abdossi V, Dehestani A, Pirdashti H, Babaeizad V (2016). Potassium phosphite affects growth, antioxidant enzymes activity and alleviates disease damage in cucumber plants inoculated with *Pythium ultimum*. Arch Phytopathology Plant Protect.

[13] Hoagland DR, Arnon DI. The water culture method for growing plants without soil. In: Arnon DI, editor. : Berkeley, College of Agriculture, University of California; 1950. p. 1–32.

[14] Araujo L, Paschoalino RS, Rodrigues FA. Phytopathology: Microscopic aspects of silicon-mediated rice resistance to leaf scald; 2016. http://dx.doi.org/10.1094/PHYTO-04-15-0109-R. 10.1094/PHYTO-04-15-0109-R 26237696

[15] Attard E. Rapid detection of Cucurbitacins in tissues and in vitro cultures of *Ecballium elaterium* (L.) A. Rich. Cucurbit Genetics Cooperative Report. 2002;25:71–5.

[16] Clinical and Laboratory Standards Institute. Methods for dilution antimicrobial susceptibility tests for bacteria that grow aerobically: Approved standard. Clinical and Laboratory Standards Institute. Wayne: CLSI publication; 2012. M7-A9.

[17] Baron EJ, Fingold SM (2007). Bailey & Scott Diagnostic Microbiology.

[18] Araujo L, Bispo WMS, Rios VS, Fernandes SA, Rodrigues FA. Induction of the phenylpropanoid pathway by acibenzolar-s-methyl and potassium phosphite increases mango resistance to *Ceratocystis fimbriata* infection. Plant Dis. 2015;99:447–59. 10.1094/PDIS-08-14-0788-RE 30699557

[19] Babu RM, Sajeena A, Samundeeswari AV, Sreedhar A, Vidhyasekeran P, Reddy MS (2003). Induction of bacterial blight (*Xanthomonas oryzae* pv. Oryzae) resistance in rice by treatment with acibenzolar-S-methyl. Ann Appl Biol.

[20] Badmanaban R (2010). Studies on anthelmintic and antimicrobial activity of the leaf extracts of *Lagenaria siceraria*. J Glob Pharma Technol.

[21] Putalun W, Luealon W, De-Eknamkul W, Tanaka H, Shoyama Y (2007). Improvement of artemisinin production by chitosan in hairy root cultures of *Artemisia annua* L. Biotechnol Lett.

[22] Chang JH, Shin JH, Chung IS, Lee HJ (1998). Improved menthol production from chitosan elicited suspension culture of *Mentha piperita*. Biotechnol Lett.

[23] Baque MA, Shiragi MHK, Lee EJ, Paek KY (2012). Elicitor effect of chitosan and pectin on the biosynthesis of anthraquinones, phenolics and flavonoids in adventitious root suspension cultures of *Morinda citrifolia* (L.). Aust J Crop Sci.

[24] Liang YC, Sun WC, Si J, Römheld V (2005). Effects of foliar- and root-applied silicon on the enhancement of induced resistance to powdery mildew in *Cucumis sativus*. Plant Pathol.

[25] Orlita A, Sidwa-Gorycka M, Paszkiewicz M, Malinski E, Kumirska J, Siedlecka EM, Łojkowska E, Stepnowski P (2008). Application of chitin and chitosan as elicitors of coumarins and fluoroquinolone alkaloids in *Ruta graveolens* L. (common rue). Biotechnol Appl Biochem.

[26] Sotiroudis G, Melliou E, Sotiroudis TG, Chinou I (2010). Chemical analysis, antioxidant and antimicrobial activity of three Greek cucumbers (*Cucumis sativus*) cultivars. J Food Biochem.

